# The Retention of Full Upper and Lower Dentures

**Published:** 1921-05

**Authors:** Russell W. Tench

**Affiliations:** New York


### THE RETENTION OF FULL UPPER AND LOWER DENTURES

BY RUSSELL W. TENCH, D.D.S., NEW YORK

A majority of our profession look upon the problem of constructing full upper and lower dentures, which will prove efficient, comfortable and satisfactory to their patients, as most vexatious. That this is so must be attributed largely to the fact that but few operators have a proper conception of the factors that govern the success or failure of their prosthetic efforts; many go about their work blindly, trusting largely to luck and the laboratory to rescue them from a bad situation, hoping that time and a kind Providence may come to the rescue by adapting the patient's mouth to their dentures, and knowing that after a time, becoming wearied, many patients will endure anything. Efficiency is often totally ignored. The easy way is followed and sometimes achieves an apparent success.

It takes but very little more time in the aggregate to construct wonderfully efficient dentures that may be worn without soreness or discomfort to the patient immediately, or within two weeks after the day they are adjusted to the mouth, than it takes to travel the usual road of hasty, imperfect methods, dissatisfaction, complaints and adjustments to the point where the patient is said to be satisfied or, more aptly, resigned to fate.

The difference between dentures that satisfy and the other kind may all be summed up in the phrase, "knowledge skillfully applied." To possess knowledge requires patient, thoughtful study. To acquire any degree of skill requires that the operator be willing to practice, to try and to try again and again patiently and with the exercise of the faculties of self-control and reason till the problem involved is mastered. It is useless to try to do a thing that can not be done—to waste effort. But that which is being done by many can be done again by any one who has the will, desire, inspiration, or whatever you may choose to call it, to keep everlastingly at a thing till he learns to do it.

I make these assertions because I know that a small proportion of the dental profession attempt to excuse themselves from performing the best service for their patients, by saying that the patient will not pay for the highest type of denture service, that the time required to become reasonably proficient in the methods that are being advocated for the construction of the highest type of dentures is too great. They can not afford it; why, bless you, the reason they can not afford it is because they won't try it. For myself, there is no satisfaction in trying to do anything but the best that is possible for any patient, and I can not bring myself to sympathize with those who would adopt a quick method or an easy method which I know in my heart is not based on principles that will make it the ultimate, the best, when carried to completion. There can be no higher professional precept than to do unto others as you would that they should do unto you.

The factors that we must understand and know before any technic of impression taking can be brought to its greatest possibilities of perfection may be resolved into two groups, viz.: those that tend to displace dentures, and those that assist in retention. There are two groups of forces that may act on a denture, either singly or together. One group helps to hold the denture in place and the other group tends to force it from its seat on the ridge. When the forces that seek to unseat the denture are just balanced by the forces that tend to retain the denture, the denture will "stay put." If we know what the displacing factors are and the limitations of these factors, we may maneuver so that these forces may only act weakly on the denture or assist in retaining it. Then having a full knowledge of the retaining factors we may utilize them to the fullest and thereby render a truly professional service to our patient which can not help but redound to our own benefit in lessening our worries and in making possible a more professional remuneration.

Preparatory to taking up the study of the retention and displacement factors just mentioned, let us consider that a denture consists of two parts, one which we will call the base and define as that portion of the upper denture that is in intimate contact with the alveolar ridge and hard palate and part of the soft palate, and that portion of the lower denture that covers the alveolar ridge. Superimposed on the base is the dental arch, which is that portion of a denture that holds the teeth and attaches them to the base.

The pressure exerted by the atmosphere in which we live is the most important single factor with which we are concerned in a study of the problem of denture retention. The retention of our denture will be good or bad in direct ratio to our utilization of this important factor. The measure in which we are able to utilize atmospheric pressure is determined by the adaptation and extension of the base of the denture in question.

Extension, as we will consider it, deals with the area that the denture base covers. Maximum extension is required if maximum retention is to be obtained. The larger the area that a denture can be made to cover, the greater the force the atmosphere can exert on it, hence the greater resistance it offers to displacing forces. A denture has reached the point of maximum extension when the peripheral portions of its base reaches to, or in some cases very slightly beyond the point of attachment of muscle fibers that bound it.

It is important to realize that full extension is necessary. Just at the point of muscle attachment the oral tissues are usually somewhat soft and thick, and at this point they must be slightly compressed to form a valve that will yield to the movement of the denture without allowing air to penetrate under the base. If this rule is not followed the displacing factors will usually overbalance the retaining factors with sad results. Very few dentures are made today which measure up to this rule. Uppers are turned with a pencil or a file largely under the guidance of fancy. Lowers are made too short and too narrow.

A second point about maximum extension worthy of consideration is, that the force of mastication is distributed over a large area and patients can employ full muscle tension when necessary to masticate hard or tough foods without discomfort.

A denture base that is intimately adapted displaces air, the weight of which exerts pressure of about fifteen pounds on every square inch of base area. The denture is literally floated into contact with the mouth tissues by the weight of the air that it displaces. Let the adaptation of the denture at its periphery be slightly imperfect, letting air leak into and fill the denture till it sinks in the air as a boat filled with water sinks in the water. Under such circumstances the denture, if it is an upper, will drop; if it is a lower it will bog around loosely in the mouth as the patient talks or eats. This illustration may seem a little academic and far-fetched, but it will serve its purpose if I can convince you that it is the pressure of the atmosphere that retains dentures. No mention has been made of vacuum, which I have intentionally ignored, because dentures can be made to resist displacement to a greater degree without so-called vacuum chambers or suction devices than is ever possible with them; also, because when you use the term suction, meaning the creation of a vacuum more or less complete, you are simply saying in another way the pressure of the atmosphere. To understand the foregoing statement is to divest the problem of retention of some of the mystery that has for a long time surrounded it. When a vacuum chamber or a relief is made in a denture, a certain amount of air is retained under the denture, and air being slightly compressed as the denture is seated exerts its expansive pressure and has a tendency to balance the external atmospheric pressure and decreases the amount of force required to displace the denture in mastication. I do not mean here to imply that a relief is not a good thing; it is necessary, but so-called suction chambers are much less potent in retention than extension and adaptation. Relief is often necessary to secure adaptation. Perfect adaptation of a base, no matter of what material it is made, is largely a matter of accident if it is attained. That this is so is largely due to expansions and contractions of the metals used in constructing metal bases, and to the same phenomena in rubber or combination rubber and metal, or metal and porcelain dentures. The imperfection of materials of which denture bases may be constructed makes it very important that we use the greatest care in all stages of denture work; that we pay every attention to securing accuracy in every detail of our work to the highest degree possible.

Minute imperfections in adaptation are overcome to a great degree by saliva and the action of capillary attraction and adhesion which hold it interposed between the denture and the mucosa, in which position it acts as a seal to keep air from penetrating under the denture. Some of us, when we have been a little too careless in our technic, have errors which even saliva will not save us from, and we resort to a thicker medium for this purpose which has to be sifted on to the denture from a can, but which lacks the advantage that saliva possesses of being replenished automatically.

The quality of adaptation and the selection of an impression material with which to obtain the best adaptation depends very largely on the condition of the mucosa covering the ridges.

Opposed to the force of atmospheric pressure and the correlated factors that make possible the maximum degree of retention are certain other factors arising from the force of muscular contraction. These factors may be grouped and called displacement factors. Their action is largely opposed to retention, and a successful denture must utilize enough of the possibilities of the retention group to offset all possible strain imposed by the displacement group; and at the same time the forces that may cause displacement must, if possible, be prevented from acting to displace the denture or be used to aid in its retention. Leverage enters largely into the consideration of this phase, and where its action is unavoidable it should be allowed to act on the upper rather than the lower denture. In all instances leverage must be reduced to the minimum permitted by esthetics, if the greatest permanence of results is expected.

The contractile force of muscle tissue acts on the periphery of the base of both the upper and lower dentures and through the dental arch.

If the base of the denture is permitted to extend beyond the point of muscle attachment to the palatal or buccolabial borders of the upper denture, a strain is created which may be sufficient to displace it. If this result is not apparent in a few days it may develop in a month or two. If the denture is retained in spite of this error soreness will result and trimming is made necessary.

To avoid displacing muscle strain and soreness the upper denture should end posteriorily at the point where the soft tissue flexes when the patient says "Ah." When the tissue covering the mouth is thick and soft or when the anterior ridge is flabby, the upper denture may safely terminate anywhere up to one-eighth of an inch posterior to this point of flexion.

The lower denture rests on a moving support, and the resulting muscle pressures are much more potent in displacing it if they are not avoided. On the lingual side of the lower ridge the periphery of the lower base should rest snugly against the tissues at or about one millimeter below the mylohyoid ridge. Impressions will invariably extend from one-eighth to a half-inch below this point and must be trimmed to the proper outline to secure the best suction. A properly-taken impression will be perfectly trimmed for the *frenum linguae.* The buccolabial flanges will require to be trimmed to the point where a vertical pull on the muscle of the lip or cheek will not unseat the denture.

Muscle contractions act on the dental arches through the bolus of food during mastication, and when their force is magnified by leverage due to incorrect design of the arches the efficiency of the dentures is greatly impaired and often entirely destroyed. By setting the lower teeth directly over the lower ridge, and as close to the ridge as ethetics will permit, the stability of the lower denture is greatly increased. Taken bucco or labiolingually the center of any given lower tooth should be directly above the middle line of the lower ridge. When the lower teeth are placed in this manner the tongue is allowed ample room, and the buccal and labial muscles are prevented from exerting dislodging pressure on them in either speech or mastication. The objection may be raised here that upper teeth will be set an unnecessary distance outside of the ridge, due to the fact that the lower ridge is usually considerably larger than the upper. This is so, but the area of the upper affords greater possibilities for retention and the upper denture rests on an immovable supporting member which more than offsets any increased leverage caused by setting the upper teeth outside of the ridge when this is necessary.—*Dental Summary*, 1921.

